# Lignin-Rich PHWE Hemicellulose Extracts Responsible for Extended Emulsion Stabilization

**DOI:** 10.3389/fchem.2019.00871

**Published:** 2019-12-17

**Authors:** Maarit H. Lahtinen, Fabio Valoppi, Venla Juntti, Sami Heikkinen, Petri O. Kilpeläinen, Ndegwa H. Maina, Kirsi S. Mikkonen

**Affiliations:** ^1^Department of Food and Nutrition, University of Helsinki, Helsinki, Finland; ^2^Faculty of Agriculture and Forestry, Helsinki Institute of Sustainability Science, University of Helsinki, Helsinki, Finland; ^3^Department of Chemistry, University of Helsinki, Helsinki, Finland; ^4^Natural Resources Institute Finland (Luke), Helsinki, Finland

**Keywords:** hemicellulose, lignin, lignin carbohydrate complex, emulsion, lipid oxidation

## Abstract

Wood hemicelluloses have an excellent capacity to form and stabilize oil-in-water emulsions. Galactoglucomannans (GGM) from spruce and glucuronoxylans (GX) from birch provide multifunctional protection against physical breakdown and lipid oxidation in emulsions. Phenolic residues, coextracted with hemicelluloses using the pressurized hot water (PHWE) process, seem to further enhance emulsion stability. According to hypothesis, phenolic residues associated with hemicelluloses deliver and anchor hemicelluloses at the emulsion interface. This study is the first to characterize the structure of the phenolic residues in both GGM- and GX-rich wood extracts and their role in the stabilization of emulsions. PHWE GGM and GX were fractionated by centrifugation to obtain concentrated phenolic residues as one fraction (GGM-phe and GX-phe) and partially purified hemicelluloses as the other fraction (GGM-pur and GX-pur). To evaluate the role of each fraction in terms of physical and oxidative stabilization, rapeseed oil-in-water emulsions were prepared using GGM, GX, GGM-pur, and GX-pur as stabilizers. Changes in droplet-size distribution and peroxide values were measured during a 3-month accelerated storage test. The results for fresh emulsions indicated that the phenolic-rich fractions in hemicelluloses take part in the formation of emulsions. Furthermore, results from the accelerated storage test indicated that phenolic structures improve the long-term physical stability of emulsions. According to measured peroxide values, all hemicelluloses examined inhibited lipid oxidation in emulsions, GX being the most effective. This indicates that phenolic residues associated with hemicelluloses act as antioxidants in emulsions. According to chemical characterization using complementary methods, the phenolic fractions, GGM-phe and GX-phe, were composed mainly of lignin. Furthermore, the total carbohydrate content of the phenolic fractions was clearly lower compared to the starting hemicelluloses GGM and GX, and the purified fractions GGM-pur and GX-pur. Apparently, the phenolic structures were enriched in the GGM-phe and GX-phe fractions, which was confirmed by NMR spectroscopy as well as by other characterization methods. The frequency of the main bonding pattern in lignins, the β-O-4 structure, was clearly very high, suggesting that extracted lignin remains in native form. Furthermore, the lignin carbohydrate complex of γ-ester type was found, which could explain the excellent stabilizing properties of PHWE hemicelluloses in emulsions.

## Introduction

The sustainable use of natural resources requires the development of new functional materials from side streams of industrial processes. Woody material is renewable biomass, which contains unexploited components that can be used for valorized products. The main components of wood are cellulose (33–51%), lignin (21–32%), and hemicelluloses (23–31%), and it contains minor amounts of other compounds, such as extractives and minerals (Fengel and Wegener, 1983; Sjöström, 1993). The processes of pulp mills are optimized and efficient for the production of cellulosic fibers from wood, which are still needed for many traditional products, such as paper and board materials (van Heiningen, 2006). New technologies are also under development for cellulosic fibers, for example as textile fibers, as reinforcing structures in composite materials, and in the form of nanocellulose (Faruk et al., 2012; Kim et al., 2015; Sixta et al., 2015). However, there is also a general need and will to produce valorized products and processes for the currently underutilized parts of wood as well as other sources of biomass, namely, hemicelluloses and lignin (Faruk et al., 2012; Sainio et al., 2013).

Hemicelluloses can be extracted from biomass prior to other processing steps by using pressurized hot water (Kilpeläinen et al., 2014). During pressurized hot water extraction (PHWE), hemicelluloses and sulfur-free lignin are released from woody material at temperatures of 160–170°C. Hemicelluloses and lignin are partially separated, and extracts enriched with hemicelluloses can be further purified from lignin using other methods, such as ultrafiltration or precipitation with ethanol, if necessary (Bhattarai et al., 2019). We have recently developed a method of using differing centrifugal forces to separate hemicellulose- and lignin-rich fractions of PHWE extracts (Valoppi et al., 2019a).

The chemical structure of wood components reflects their material properties. The role of hemicelluloses is to provide flexibility to the cell wall. In contrast to cellulose, hemicelluloses and lignin are heterogeneous materials with a complex chemical structure, which is further dependent on the type of wood (Sjöström, 1993). Thus, the main hemicelluloses in softwoods and hardwoods are glucomannans and glucuronoxylans, respectively, although softwoods also contain minor amounts of glucuronoxylans and hardwoods have some glucomannans (Sjöström, 1993).

In spruce, the main hemicelluloses are galactoglucomannans (GGM) (Lundqvist et al., 2002; Hannuksela and Hervé du Penhoat, 2004; Xu et al., 2007). The backbone of GGM consists of (1 → 4)-linked β-D-mannopyranosyl (Man*p*) units and (1 → 4)-linked β-D-glucopyranosyl (Glc*p*), and some α-galactopyranosyl (Gal*p*) units are (1 → 6)-linked to the backbone as single-unit side groups. The proportions of the carbohydrates in GGM are 4:1:0.5 for Man*p*:Glc*p*:Gal*p* (Willför et al., 2003). In birch, the main hemicelluloses are 4-O-methylglucuronoxylans (GX) (Teleman et al., 2002). The backbone of GX consists of (1 → 4)-linked β-D-xylopyranosyl (Xyl*p*) units, to which α-4-O-methylglucuronic (MeGlcA) substituents are (1 → 2)-linked. The proportions are 0.5–1:10 for MeGlcA:Xyl*p* (Teleman et al., 2002). Both GGM and GX contain O-acetyl (OAc) groups at C-2 and/or C-3 positions of Man*p* and Xyl*p* units. The reported degrees of acetylation are about 15–20% for spruce GGM and 25% for birch GX (Lundqvist et al., 2002; Xu et al., 2007; Du et al., 2013).

Lignin is an aromatic polyphenol composed of phenylpropane units (Sjöström, 1993; Boerjan et al., 2003; Vanholme et al., 2010). The role of lignin in plants is to provide mechanical strength, enable efficient liquid transportation, and provide protection against microbial attack (Fengel and Wegener, 1983; Boudet, 2000). The building blocks of lignin are the monolignols *p*-coumaryl alcohol (minor component of wood plants), coniferyl alcohol (major component of softwoods), and sinapyl alcohol (major component of hardwoods). During lignin biosynthesis, the monolignols are oxidized to form phenoxy radicals, which leads to radical polymerization and the formation of different types of bonding patterns. The most frequent structure in lignin is the β-aryl ether type (β-O-4), which is prone to react with different chemicals, leading to degradation of lignin, for example in kraft pulping process (Sjöström, 1993). The other frequent linkage types include phenyl coumaran type (β-5) and resinol type (β-β).

Hemicelluloses and lignin are closely associated in the wood cell wall. In addition to non-covalent interactions, the presence of covalently bound lignin-carbohydrate complexes (LCCs) was suggested decades ago (Fengel and Wegener, 1983). However, even today, unequivocal proof of different types of LCCs is lacking, because these structures have low frequencies and because structural modifications may occur during their isolation for characterization (Giummarella et al., 2019). Recently, isolation and characterization of the α-ether type LCC was successfully performed (Nishimura et al., 2018) and was made possible by the development of a methodology for enriching LCCs and characterizing by nuclear magnetic resonance (NMR) spectroscopy. To date, the structure of phenolic residues and their associations with hemicelluloses in PHWE extracts are largely unknown.

Hemicellulose- and phenolic-rich PHWE extracts exhibit excellent emulsifying ability and physical emulsion stabilization capacity, which gives them great potential in both bulky and specialized industrial applications, such as food, paints, cosmetics, and pharmaceuticals (Mikkonen et al., 2016, 2019; Valoppi et al., 2019b). Furthermore, PHWE hemicelluloses containing phenolic co-components offer excellent oxidative stability in rapeseed oil-in-water emulsions (Lehtonen et al., 2016, 2018). For comparison, emulsions prepared from rapeseed oil, which has been purified from natural antioxidants of the oil, tocopherols, and stabilized with Tween 20 or gum Arabic, are oxidized in a few days (Heinonen et al., 1997; Lehtonen et al., 2016). The presence of tocopherols retards the oxidation, which is, however, modest compared to PHWE GGM, which improves the oxidative stability for several weeks in an accelerated storage test (Lehtonen et al., 2018). The strong, previously developed hypothesis was that phenolic residues associated with hemicelluloses would be responsible for improved physical and oxidative stability, but their exact chemical structure was still unclear (Lehtonen et al., 2018).

The aim of this study was to reveal the structure of phenolic residues responsible for the efficient emulsion stabilization capacity of PHWE spruce GGM and birch GX. In this study, aqueous GGM and GX were centrifuged in a parallel manner to separate hemicellulose-rich supernatants and lignin-rich pellets. For the first time, the fractions were characterized in detail using complementary chemical analyses to investigate the role of the lignin-rich fraction in the physical and oxidative stability of emulsions. Furthermore, characterization of both GGM and GX using various methods enabled a comparison of softwood and hardwood hemicelluloses and different analytical methods. The results explain the structural elements in hemicellulose-rich wood extracts that are responsible for their excellent performance in emulsions.

## Materials and Methods

### Hemicelluloses

A pressurized hot water flow-through extraction (PHWE) system was used to obtain GGM-rich extract from spruce and GX-rich extract from birch (Kilpeläinen et al., 2014). Spruce sawdust (from Herralan Saha, Finland, 96.9 kg, 43.5 kg on dry basis) was extracted at 170°C for 60 min at a rate of 20 l min^−1^, and 1,000 l of the extract was collected. Birch sawdust (from Haka-Wood, Finland, 103.7 kg, 54.2 kg on dry basis) was extracted at 170°C for 60 min at a rate of 20 l min^−1^, and 700 l of the extract was collected. Both the spruce and birch extracts were ultrafiltrated to obtain concentrated extracts, as described previously (Bhattarai et al., 2019). The concentrated extracts were finally spray dried to powdered form using a Buchi Mini Spray Dryer B-290 (Buchi, Switzerland), which has the evaporation capacity of 1 l h^−1^ for water. The conditions for the spray drying used were as follows: inlet temperature 170°C, outlet temperature 65°C, and drying air flow rate 667 l h^−1^. The moisture contents of the materials were 6.0% for GGM and 3.9% for GX after storage in the dark at room temperature.

### Reagents

Rapeseed oil (Keiju, Bunge Finland Ltd, Raisio, Finland) was purchased in a local supermarket. Monosaccharides used for the analysis of carbohydrate content were L(+)-arabinose (Ara), D(+)-xylose (Xyl), D(+)-galactose (Gal), D(+)-glucose (Glc), D(+)-mannose (Man) from Merck (Darmstadt, Germany), L(+)rhamnose monohydrate (Rha) from Sigma (St. Louis, MO, USA), D(+)-sorbitol, D(+)-galacturonic acid monohydrate (GalA) from Fluka (St. Louis, MO, USA), and D(+)-glucuronic acid sodium salt monohydrate (GlcA) from Aldrich. The reagents used for silylation were bis(trimethylsilyl)trifluoroacetamide (BSTFA) from Merck (Darmstadt, Germany) and trimethylsilyl chloride (TMSCl) from Fluka (St. Louis, USA). Pullulan kit standards (Z-POS-pulkith) were used for size-exclusion chromatography (SEC; Postnova Analytics, Landsberg am Lech, Germany). Vanillin (Sigma-Aldrich) and syringaldehyde (Aldrich) were used for the quantification of phenols.

The solvents used for NMR analysis were D_2_O and d_6_-DMSO, which were purchased from Eurisotop (Saint-Aubin, France). All other solvents used were HPLC or LC-MS grade. Milli-RO water was used in centrifugal separation, and Milli-Q was used as a solvent in chemical analysis.

### Centrifugation of Hemicelluloses

The 10% solutions of GGM and GX were dissolved in Milli-RO water and stirred for 2 h at room temperature (total amount 150 ml). The solutions were then centrifuged at 18,677 g at room temperature for 20 min. The supernatants (GGM-pur and GX-pur) and pellets (GGM-phe and GX-phe) were collected and freeze-dried separately. The recovered yields were 91.0% for GGM-pur, 4.6% for GGM-phe, 86.6% for GX-pur, and 5.8% for GX-phe (based on dry material).

### Purification of Rapeseed Oil

Rapeseed oil (Keiju, Bunge Finland Ltd, Raisio, Finland) was purchased from a supermarket and stripped of tocopherols using a previously described method (Lampi et al., 1999). The composition has been determined in earlier publications (for example Lehtonen et al., 2016, 2018).

### Preparation of Emulsions

The amount of oil and emulsifier, and the applied buffer and pH used, were based on optimizations performed in previous studies (Mikkonen et al., 2016). Emulsions containing hemicelluloses (1 w-%), GGM or GX, or their supernatants, GGM-pur and GX-pur, in 25 mM Na-citrate buffer, pH 4.5, and stripped rapeseed oil (5 w–%) were prepared by high-pressure homogenization using a previously described method with some modifications (Lehtonen et al., 2016). The total weight of each emulsion was 80 g. First, carbohydrate was dissolved in buffer by stirring overnight at room temperature. After the addition of oil, the coarse emulsion was prepared by stirring the resulting mixture with Ultra-Turrax (T25 basic, IKA, Staufen, Germany) at 22,000 rpm for 2 min. The mixture was further homogenized by passing it continuously through a high-pressure homogenizer for 32 s at a pressure of 800 bar (Microfluidizer 110Y, Microfluidics, Westwood, MA, USA). The homogenizer was configured with 75 μm Y-type F20Y and 200 μm Z-type H30Z chambers in series.

### Accelerated Storage Test

For the accelerated storage test, emulsions were stored in glass bottles (100 ml) at 40°C in the dark for 3 months. For all the determinations, emulsions were gently mixed by turning their containers upside down 10 times before sampling. The properties of emulsions were monitored on the day of preparation, after 1 and 2 weeks of preparation, and after that approximately every 2 weeks. The properties, which were monitored during the storage test, were droplet-size distribution and peroxide value. At the end of the storage period, optical microscopy was used to visualize the morphology of emulsions (AxioScope A1, Carl Zeiss Inc., 203 Oberkochen, Germany). For microscopic imaging, the 100x objective was used, with a Zeiss Phase Contrast condenser with a Ph3 port.

### Droplet-Size Distribution

The droplet-size distribution was determined by static light scattering technique using a Mastersizer Hydro 3000 (Malvern Instruments Ltd, Worcestershire, UK). The refractive indexes used were 1.33 for water and 1.47 for rapeseed oil (Rumble, 2018–2019). The emulsions were added directly into the dispersion accessory, which allowed dilution to avoid multiple scattering effects. The rotor speed during measurement was 2,400 rpm. Each sample was measured three times.

### Determination of Peroxide Value

Peroxide values (PVs), as an indicator of primary oxidation of emulsions, were determined by a previously reported method, in which lipids are first released and extracted and then analyzed using the ferric thiocyanate method (Lehtonen et al., 2011, 2016). Analytical samples of extracted lipids were prepared in duplicate, and from both samples of extracted lipids, two samples were withdrawn for the determination of PVs. Thus, the results were calculated as averages and standard deviations of four measured values.

### Quantitative Analysis of Carbohydrates

The carbohydrate content of GGM and GX and of their centrifuged fractions, GGM-pur, GGM-phe, GX-pur, and GX-phe, were analyzed by GC using the acid methanolysis and silylation method described previously (Sundberg et al., 1996). The instrumental details of the analysis were described previously as well (Chong et al., 2013). External calibration of five levels of concentration was used to calculate the amount of each monosaccharide in the samples. Methyl glucuronic acid (MeGlcA) was quantified based on the two major signals and the D-glucuronic acid standard (Chong et al., 2013). All samples were analyzed in triplicate (*n* = 3).

### Structural Characterization of Starting Materials GGM and GX (Non-acetylated) and Phenolic Samples GGM-Phe and GX-Phe (Acetylated) by 2D HSQC and HSQC-TOCSY NMR, and Evaluation of Diffusion Constants by 2D DOSY NMR (Acetylated or Partially Acetylated Samples)

For structural characterization of the starting hemicelluloses, GGM and GX, and the fractions enriched with phenolic compounds, GGM-phe and GX-phe, 2D Heteronuclear Single Quantum Coherence (HSQC) spectra, 2D Heteronuclear Single Quantum Coherence—Total Correlation SpectroscopY (HSQC-TOCSY) spectra and 2D Diffusion Ordered SpectroscopY (DOSY) data were acquired using a Varian Unity Inova 500-MHz spectrometer equipped with a 5-mm pulsed-field-gradient triple resonance probehead (^1^H, ^13^C, ^15^N) capable of delivering z-gradient amplitudes up to 20 G/cm. The pulse sequences used in this study were readily available in Varian VNMR 6.1C spectrometer operating software.

All samples were analyzed in DMSO-d_6_ at 27°C. Of the starting hemicelluloses, GGM and GX, 30 mg was first dispersed in D_2_O (0.7 ml), freeze-dried, and finally dissolved in DMSO-d_6_ (0.7 ml). Of the phenolic fractions, GGM-phe and GX-phe, 40 mg was acetylated in pyridine/acetic anhydride (1:1), and the remaining reagents/solvents were removed by evaporating the mixture with ethanol twice, with toluene four times, and finally with chloroform under reduced pressure twice. The acetylated sample was then dissolved in DMSO-d_6_ (0.7 ml) and analyzed.

All HSQC spectra were recorded with a standard, phase-sensitive, gradient-selected HSQC sequence using echo-antiecho acquisition mode in the indirectly detected dimension. The hard, rectangular 90° pulse widths were 6.7 and 11.5 μs for ^1^H and ^13^C, respectively. The spectral width was 5,573 Hz for ^1^H (carrier at 5.47 ppm) and 25,133 Hz for ^13^C (carrier at 90.01 ppm). The relaxation delay was 1 s, and the acquisition time was 0.128 s. Experiments were acquired using 64 steady-state scans, 64 transients, and a data matrix size of 713 (^1^H, complex points) × 200 (^13^C, complex points). The data matrices were apodized by a Gaussian function (gf = 0.032) in ^1^H-dimension and a Gaussian function (gf = 0.004) in ^13^C-dimension and zero-filled up to 1,024 (^1^H, complex points) x 1,024 (^13^C, complex points) prior to Fourier transformation.

For HSQC-TOCSY, a standard phase-sensitive, gradient-selected (echo-antiecho) pulse sequence was applied. The hard, rectangular 90° pulse widths were 6.7 and 11.5 μs for ^1^H and ^13^C, respectively. The spectral width was 5,573 Hz for ^1^H (carrier at 5.47 ppm) and 25,133 Hz for ^13^C (carrier at 90.01 ppm). Relaxation delay was 1 s and acquisition time was 0.184 s. The TOCSY mixing was performed using the windowed MLEV-17 spin-lock scheme (Griesinger et al., 1988) to suppress possible ROESY correlations. TOCSY mixing was applied for 100 ms at an RF power of 7.9 kHz. Experiments were acquired using 64 steady-state scans, 64 transients, and a data matrix size of 1,024 (^1^H, complex points) × 200 (^13^C, complex points). The data matrices were apodized by a Gaussian function (gf = 0.037) in ^1^H-dimension and a Gaussian function (gf = 0.003) in ^13^C-dimension and zero-filled up to 1,024 (^1^H, complex points) × 1024 (^13^C, complex points) prior to Fourier transformation.

2D DOSY spectra were measured using Bipolar Pulse Pair Stimulated Echo sequence with convection compensation (BPPSTE-cc) (Wu et al., 1995; Jerschow and Müller, 1997). The spectral width of 8,000 Hz in ^1^H-dimension was covered by the acquired 8,002 complex points, resulting in 1-s acquisition time. The relaxation delay was 1 s. In order to map diffusion coefficients (D_c_), 20 spectra were acquired with increasing amplitudes of rectangular diffusion gradient pulses (from 0.5 to 20 G/cm). The diffusion gradient pulse duration was 2 ms, and the diffusion delay was 600 ms. The eddy-current recovery delay was 150 μs. A total of four steady-state scans and 32 transients were used to collect all 20 of these spectra. The free induction decays (FIDs) were apodized using an exponential weighting function (10-Hz line broadening) and zero-filled up to 8,192 complex points before the Fourier transform. The 2D DOSY plots were calculated using the dosy macro (a monoexponential fit on the peak tops) incorporated into VNMR 6.1C software. The final size of the diffusion dimension in 2D DOSY was 256 data points. The diffusion coefficients of the macromolecule and the residual DMSO signal were estimated from each DOSY spectrum (see Figures S2A–D); the horizontal line shows the estimated values for D_c_(GGM), D_c_(GGM-phe), D_c_(GX), and D_c_(GX-phe). Moreover, the D_c_(DMSO) value for each sample is shown. In order to compensate the effects of possible sample viscosity differences in the diffusion coefficient results, the estimated diffusion coefficients of the macromolecules were corrected using the measured diffusion coefficient values for residual DMSO signal of the solvent (Kavakka et al., 2009). In the correction procedure, the D_c_(DMSO) value in the GGM sample was selected as the reference [D_c_(DMSO_ref_)], and the DOSY results for each sample were multiplied by D_c_(DMSO_ref_)/D_c_(DMSO); that is, after the correction, D_c_(DMSO) was the same for all four 2D DOSY spectra.

### Analysis of Molar Masses

Molar masses of GGM, GX, and their centrifuged fractions were analyzed by SEC (GPCmax, Viscotek Corp., Houston, TX, USA). The instrumental details were described in a previous study (Pitkänen et al., 2011). The samples were dissolved in 0.01 M LiBr in DMSO overnight to a concentration of 10 mg ml^−1^ and filtered through a 0.45 μm syringe filter (GHP Acrodisc 13 mm, Pall Corp., Ann Arbor, MI, USA). The volume of the sample injected was 100 μl. DMSO, containing 0.01 M LiBr, was used as the eluent, with a flow rate of 0.8 ml min^−1^. The molar mass of samples was estimated using pullulan standards for calibration (342, 1,320, 5,900, 11,800, and 22,800 Da). The elution data were processed using the OmniSEC 4.5 software (Viscotek Corp.).

### Determination of Phenolic Content by Pyrolysis GC-MS

Pyrolysis of the starting hemicellulose samples GGM and GX and the centrifuged pellets GGM-phe and GX-phe was performed using a foil pulse-type Pyrola 2000 MultiMatic pyrolyzer (Pyrol AB, Lund, Sweden). The pyrolysis unit was connected to an Agilent GC model 7890B equipped with an HP5-MS column (25 m x 0.20 mm, film thickness 0.33 μm), coupled with an Agilent 5977B quadrupole-MSD with EI ionization (Agilent Technologies, Santa Clara, CA, USA). Approximately 100 μg of dry sample and a drop of acetone was applied to the Pt filament and pyrolyzed at 600°C for 2 s. Conditions for the GC analysis were as follows: gas flow (helium) 0.8 ml min^−1^, injector temperature 300°C, split 1:20; the column oven temperature was 50°C for 1 min, then heated with a rate of 8°C min^−1^ to 320°C, which was maintained for 5 min; the transfer line temperature was 250°C.

Compounds were identified by comparing acquired spectra with spectra in the Laboratory of Wood and Paper Chemistry, Åbo Akademi, Finland (own database) and with Wiley 10th/NIST2012. The results were calculated as the relative abundance of each pyrolysis product (peak area-% of total peak area).

### Quantitative Determination of Extractable Phenolic Residues by UHPLC-DAD-FLD and Identification by LC-MS

Phenolic residues of the pellets were extracted and quantified based on a previously described method, which was slightly modified (Lehtonen et al., 2016). For extraction, 10 mg of GGM-phe or GX-phe was dissolved in 80% ethanol (1 ml) and centrifuged three times. The supernatants were combined and evaporated under reduced pressure. The ethanol-soluble phenolic residues were then extracted with ethyl acetate (3 × 500 μl), after adjusting the pH by adding 400 μl of 6 M HCl, and finally the ethyl acetate was evaporated under nitrogen stream. The ethanol-soluble phenolics were analyzed after extraction (neutral) or after acid or base hydrolysis. The pellets containing remaining carbohydrates from GGM-phe were also hydrolyzed after extraction with acid or base, as described previously (Lehtonen et al., 2016). No pellet remained from GX-phe after extraction of phenol. All treatments were performed in triplicate (*n* = 3).

For the analysis, all samples were redissolved in 10% MeOH (1 ml), filtered through a 0.2-μm PTFE syringe filter (VWR International, Radnor, PA, USA), and separated with an ACQUITY UPLC system (Waters, Milford, MA, USA), as described previously (Kylli et al., 2011; Lehtonen et al., 2016). The injection volume for all samples was 10 μl. In addition, the sample containing the ethanol-soluble phenols hydrolyzed with base was diluted to 1/10 for quantification at the concentration levels explained below.

For the identification of the main phenolic compounds extracted, the same UPLC system equipped with a Waters Synapt G2-S*i* high definition mass spectrometer with a LockSpray Exact Mass Ionization Source was used. The LC-MS spectra were processed with MassLynx 4.1 software, which uses an *m/z* lockmass value of 556.2771 for leucine eukephalin (equal to *m/z* of [M+H]^+^ + e^−^). Based on identification with MS, the main phenolic compounds extracted were quantified using vanillin and syringaldehyde as the reference compounds, with three levels of concentration in the range of 10–45 ng/injection. LC-MS spectra are presented in the Supplementary Material. ESI-MS vanillin: *m/z* 153.0552 [M + H]^+^ (C_8_H_9_O_3_ + e^−^ requires 153.0552), syringaldehyde *m/z* 183.0657 [M + H]^+^ (C_9_H_11_O_4_ + e^−^ requires 183.0657).

## Results and Discussion

### Fractionation of Hemicelluloses and Preparation of Emulsions

The starting hemicelluloses, spray-dried hot-water-extracted GGM and GX, were fractioned by centrifugation. As will be explained in the characterization of materials, supernatants consisted of hemicelluloses partially purified from phenolic compounds (GGM-pur and GX-pur fractions), and pellets contained mainly lignin and other phenolic residues (GGM-phe and GX-phe). This solvent-free fractionation method takes advantage on the low solubility of lignin in water, which enables partial separation of precipitated lignin and water-soluble hemicelluloses (Valoppi et al., 2019a). In a recently published study (Valoppi et al., 2019a), the effect of using different centrifugal treatments on the degree of purification and properties of GGM-rich PHWE extracts was evaluated in detail. In the present study, we used high centrifugal forces, optimized in the previous study (Valoppi et al., 2019a), on both GGM and GX to compare softwood and hardwood hemicelluloses for the first time by this fractionation method and to reveal the structure of phenolic residues coextracted with hemicelluloses.

Oil-in-water emulsions were then prepared from rapeseed oil stripped from tocopherols, using the starting hemicelluloses (GGM and GX) and the purified fractions (GGM-pur and GX-pur) as emulsifiers. The resulting emulsions were characterized to investigate the effect of removed phenolic residues on the physical and oxidative stability of emulsions. During the accelerated storage test of emulsions, the droplet-size distribution was measured periodically to monitor the physical stability, and the morphology was further confirmed by microscopy.

### Physical Properties and Stability of Emulsions

**Figure 1:**
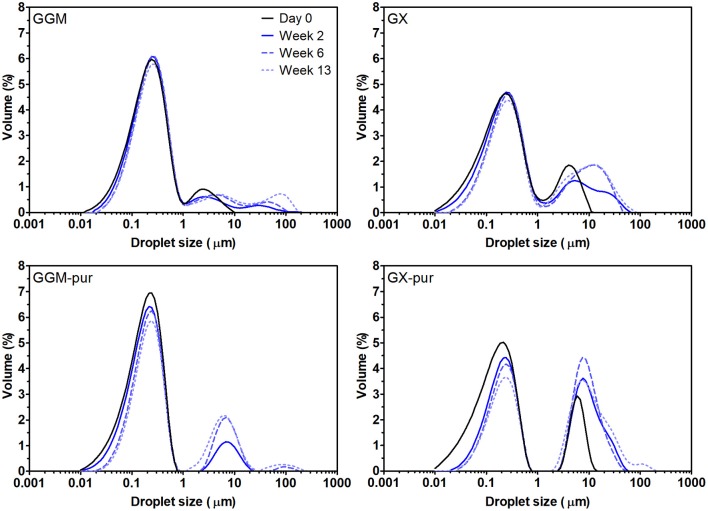
Droplet-size distribution of emulsions using the starting hemicelluloses, GGM and GX, and purified fractions, GGM-pur and GX-pur, as the emulsifiers. The physical stability of emulsions was observed during the storage test at 40°C by measuring droplet-size distribution periodically. The values for selected measurements are presented in Table 1.

The droplet-size-distribution curves of all emulsions are presented in Figure 1, and values from selected time points of measurements are presented in Table 1 (All other values for droplet-size measurements are found in Table S1). Only the fresh emulsion stabilized with GGM-pur had unimodal droplet-size distribution, and the more bimodal distribution observed previously for GX emulsions (Mikkonen et al., 2016) was most evident for fresh emulsion stabilized with GX-pur. The surface average droplet size D[3,2] for all fresh emulsions was in the range of 120–150 nm, which is similar to the previous result for PHWE GGM (Lehtonen et al., 2018). However, the D[3,2] value of GGM-pur and GX-pur (120 nm) emulsions was smaller than that of emulsions with the starting GGM and GX (140–150 nm). The more pronounced bimodal droplet-size distribution of GX-pur compared to GX emulsion also increased the volume average droplet size D[4,3], which is affected more by the larger droplets compared to D[3,2]. During the storage test, the droplet size increased for all samples, and the change was more apparent for the emulsions stabilized with the purified hemicelluloses GGM-pur and GX-pur. This was most clearly observed in the D90 values, which take into account 90% of the oil droplets, which are equal or smaller than D90. In conclusion, it seems that the fraction that was removed from both of the purified hemicelluloses GGM-pur and GX-pur by centrifugation was responsible for a slightly larger droplet size of fresh emulsions, but on the other hand, it enhanced the long-term physical stability of emulsions, in agreement with previously published results for GGM (Valoppi et al., 2019a).

**Table 1:** Average droplet-size values for fresh o/w emulsions.

**Emulsifier**	**Storage time**	**D[3,2] (μm)**	**D[4,3] (μm)**	**D10 (μm)**	**D50 (μm)**	**D90 (μm)**
GGM	Fresh	0.152	0.552	0.070	0.237	1.050
	2 weeks	0.176	1.970	0.081	0.255	1.820
	3 months	0.212	6.950	0.095	0.294	11.50
GGM-pur	Fresh	0.117	0.215	0.056	0.187	0.415
	2 weeks	0.144	1.130	0.068	0.214	4.180
	3 months	0.226	4.870	0.100	0.295	8.850
GX	Fresh	0.137	1.070	0.057	0.259	4.020
	2 weeks	0.171	3.100	0.073	0.301	9.440
	3 months	0.260	5.350	0.104	0.413	17.90
GX-pur	Fresh	0.116	1.400	0.048	0.205	6.160
	2 weeks	0.263	5.670	0.103	0.402	16.80
	3 months	0.343	9.580	0.122	3.940	24.60

*The D[3,2] and D[4,3] are the surface and volume weighted mean diameters. The D10, D50, and D90 values mean, respectively, that 10, 50, and 90% of droplets are smaller compared to that value*.

**Figure 2:**
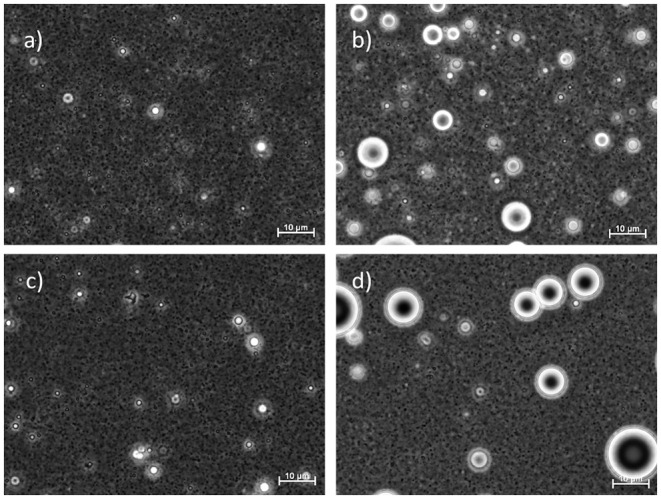
Light microscope images of emulsions at the end of the 3-month storage period. The emulsifiers are in **(a)** starting GGM, **(b)** starting GX, **(c)** GGM-pur, and **(d)** GX-pur. The length of the scale bar is 10 μm in all images.

The microscopic images obtained at the end of the 3-month storage period (Figure 2) confirm the results from the droplet-size-distribution measurements. The average droplet size D[3,2] for GGM and GGM-pur was still fairly low, 210–220 nm, at the end of the storage period, and these droplets were hardly visible by optical microscopy with the magnification used. In the image of GGM-pur in Figure 2c, there are possibly a couple of larger droplets compared to the image of GGM in Figure 2a. In the case of GX and GX-pur (Figures 2b,d), the difference in the number and size of larger droplets was more evident and clearly reflects the droplet-size-distribution data.

### Oxidative Stability of Emulsions

**Figure 3:**
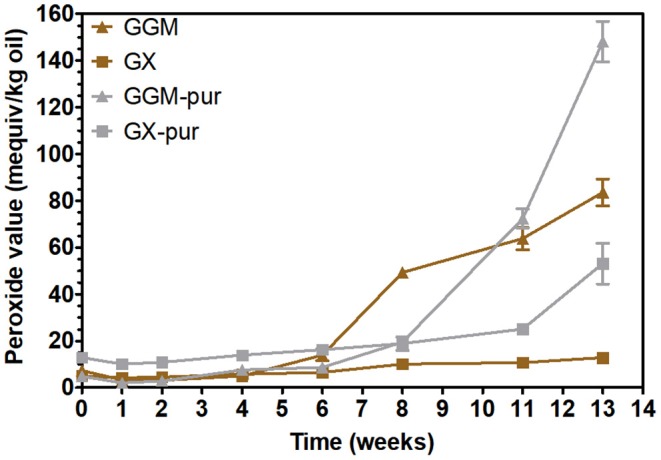
Peroxide values for rapeseed oil in emulsions using starting GGM and GX and using purified GGM-pur and GX-pur as the emulsifiers.

In order to observe the oxidative stability of emulsions, their peroxide values were measured periodically during the accelerated storage test. The oxidative stability of emulsions stabilized with GX was then investigated for the first time. Peroxide values indicate the formation of hydroperoxides, the initial oxidation products of rapeseed oil (Lehtonen et al., 2011, 2016). The results clearly show (Figure 3) that the phenolic fraction removed from the starting hemicelluloses GGM and GX was responsible for inhibiting lipid oxidation in emulsions.

The peroxide values for all emulsions were practically unchanged during the first 6 weeks of storage, which is compatible with the previously published results for concentrated PHWE GGM (Lehtonen et al., 2018). The peroxide values of all emulsions started to increase after 6 weeks, but the extent of oxidation was different at the end of the storage period. The starting hemicellulose GX was the most stabilizing of all the emulsifiers tested, because the increase of peroxide values during the 3-month storage period was fairly modest compared to other emulsifiers, although the stabilization of 6 weeks for GGM is also a notable result.

### Carbohydrate Composition of Starting Hemicelluloses and Fractionated Materials From Centrifugation

The carbohydrate composition of the starting hemicelluloses, GGM and GX, and their purified (pur) and phenolic (phe) fractions (Table 2) was analyzed in order to evaluate both the total amount of carbohydrates in each fraction and possible differences in carbohydrate composition. The total amount of carbohydrates was around 735 mg g^−1^ for GGM and 615 mg g^−1^ for GX, which is in agreement with results previously obtained for spray-dried PHWE GGM and GX (Mikkonen et al., 2019). The total carbohydrate contents for the purified fractions were 845 and 621 mg g^−1^, implying that the fractionation method increased the ratio of carbohydrates for GGM-pur but was very similar when starting GX and GX-pur were compared. For the fractions GGM-phe and GX-phe, the amount of carbohydrates was clearly lower: 251 and 181 mg g^−1^. This result indicates that 75–82% of these fractions are of an origin other than carbohydrates, which in the case of wood-based hot-water-extracted material is most likely composed of lignin or other phenolic residues.

**Table 2:** Carbohydrate composition of starting GGM and GX, and the centrifugated fractions GGM-phe and GX-phe obtained from quantitative GC analysis of acid methanolyzed and silylated samples.

**Sample**	**Ara**	**Xyl**	**Rha**	**Gal**	**Glc**	**Man**	**GalA**	**MGA**	**Total**
GGM	9.4 ± 0.2	74.5 ± 2.2	7.1 ± 0.1	54.0 ± 1.2	102.9 ± 2.4	441.2 ± 10.3	20.4 ± 0.8	25.2 ± 2.1	734.7 ± 17.7
	***2.1***	***16.9***	***1.6***	***12.2***	***23.3***	***100.0***	***4.6***	***5.7***	***166.5***
GGM-pur	9.9 ± 0.1	84.7 ± 1.5	7.3 ± 0.1	64.2 ± 2.9	117.7 ± 3.9	509.6 ± 4.3	23.6 ± 0.5	27.5 ± 1.3	844.6 ± 21.27
	***1.9***	***16.6***	***1.4***	***12.6***	***23.1***	***100.0***	***4.6***	***5.4***	***165.7***
GGM-phe	6.1 ± 0.4	22.1 ± 7.8	5.2 ± 0.4	17.8 ± 6.3	46.1 ± 9.2	137.6 ± 45.0	5.7 ± 3.7	10.7 ± 4.3	251.3 ± 75.8
	***4.4***	***16.1***	***3.8***	***13.0***	***33.5***	***100.0***	***4.1***	***7.8***	***182.7***
GX	2.2 ± 0.1	495.9 ± 3.2	7.7 ± 0.1	13.5 ± 0.3	13.3 ± 0.2	18.1 ± 0.1	11.2 ± 0.1	52.8 ± 0.4	614.8 ± 3.5
	***0.5***	***100.0***	***1.6***	***2.7***	***2.7***	***3.7***	***2.3***	***10.7***	***124.0***
GX-pur	2.4 ± 0.1	499.7 ± 14.4	7.7 ± 0.2	13.7 ± 0.6	13.0 ± 0.6	18.5 ± 0.4	11.8 ± 0.2	54.5 ± 1.2	621.4 ± 15.4
	***0.5***	***100.0***	***1.6***	***2.8***	***2.6***	***3.7***	***2.4***	***10.9***	***124.4***
GX-phe	1.6 ± 0.4	132.3 ± 11	5.0 ± 0.1	5.1 ± 0.0	6.0 ± 0.3	6.4 ± 0.3	4.5 ± 0.5	20.5 ± 2.4	181.3 ± 13.7
	***1.2***	***100.0***	***3.8***	***3.9***	***4.5***	***4.9***	***3.4***	***15.5***	***137.1***

*The results presented in normal font are expressed as mg/g of dry sample. For results presented in bold and italic font, the results have been normalized by setting a value of 100 for the main carbohydrate in the sample (i.e., for GGM, Man = 100, and for GX, Xyl = 100). Glucuronic acid (GlcA) was not detected*.

The carbohydrate composition of the starting materials and purified fractions was more similar to that of the phenolic-rich fractions. Furthermore, certain carbohydrates seemed to be associated more closely with the phenolic fractions. In both GGM-phe and GX-phe, the presence of Ara*f*, Rha*p*, Glc*p*, and MeGlcA was pronounced, even taking into account the high standard errors in the results. Different types of lignins are known to be associated with certain carbohydrates: glucomannan-lignin complexes have been isolated mainly from softwoods, whereas glucan-lignins have been found in hardwoods and xylan-lignins in both softwoods and hardwoods (Lawoko et al., 2005; Li et al., 2011; Du et al., 2013; del Río et al., 2016). Further separation and characterization of the different types of carbohydrate-lignins were beyond the scope of this work, and thus any clear conclusions about the identity of potentially different polysaccharides associated with lignin cannot be made at this point.

### Structural Characterization of Starting Hemicelluloses GGM and GX and Precipitated Fractions GGM-Phe and GX-Phe by 2D HSQC NMR Spectroscopy

**Figure 4:**
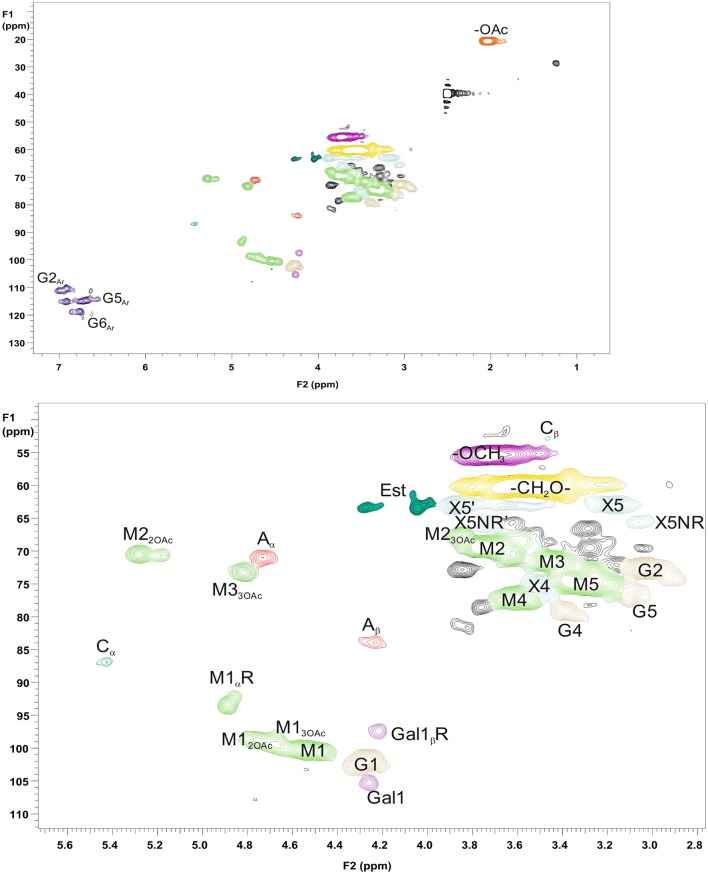
2D HSQC NMR spectrum of PHWE GGM starting material. Upper figure is the entire NMR spectrum, and lower figure is a magnification of the side-chain area. The nonacetylated sample was characterized dissolved in d_6_-DMSO. The symbols and chemical structures are presented and explained in Figure 7.

**Figure 5:**
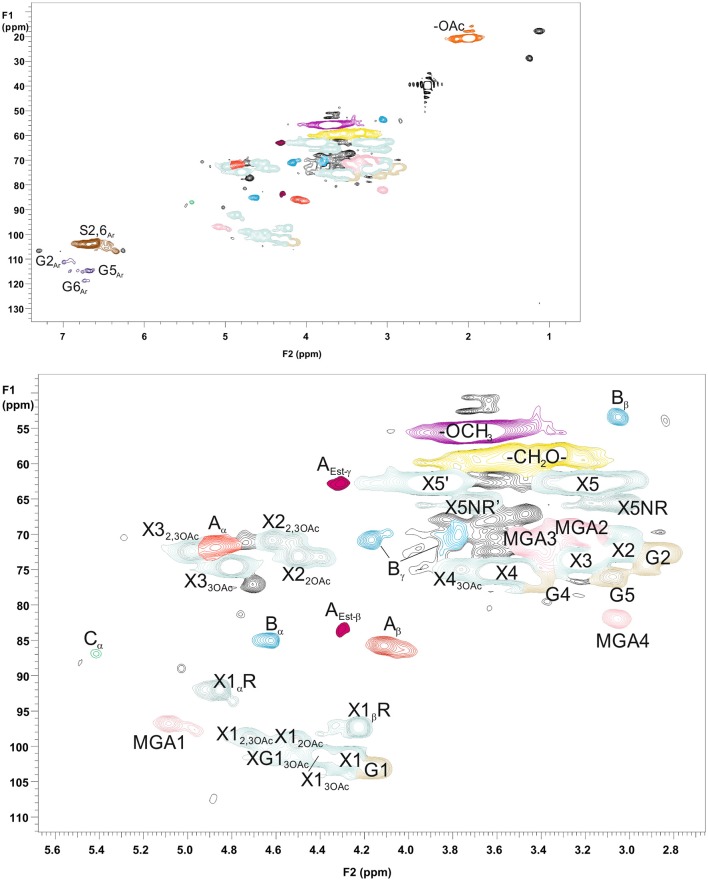
2D HSQC NMR spectrum of PHWE GX starting material. Upper figure is the entire NMR spectrum, and lower figure is a magnification of the side-chain area. The nonacetylated sample was dissolved in d_6_-DMSO for analysis. The symbols and chemical structures are presented and explained in Figure 7.

**Figure 6:**
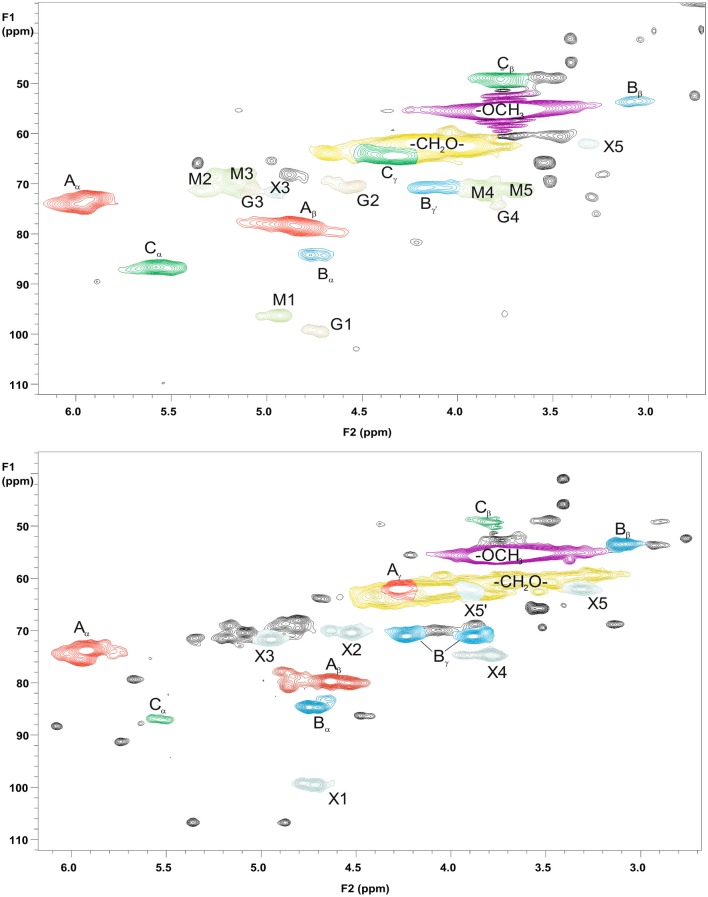
2D HSQC NMR spectrum of the fractions GGM-phe (upper figure) and GX-phe (lower figure). Only magnification of the side-chain areas is shown. The acetylated samples were dissolved in d_6_-DMSO for analysis. The symbols and chemical structures are presented and explained in Figure 7.

**Figure 7:**
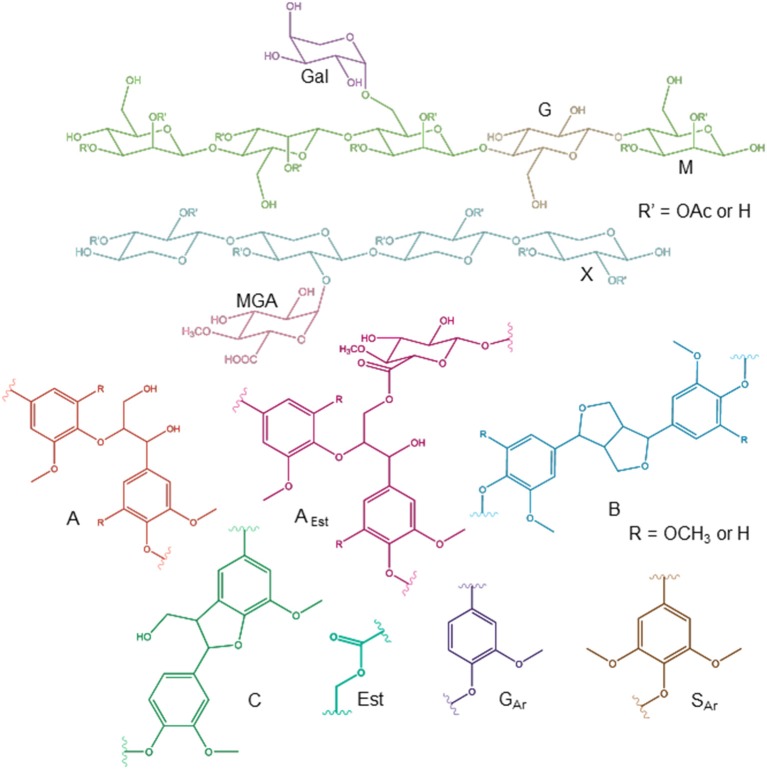
Chemical structures identified by NMR. The symbols and abbreviations used in NMR spectra are G (Glc), M (Man), MGA (MeGlcA), X (Xyl), A (β-O-4/β-aryl ether), B (β-β/resinol), C (β-5/phenyl coumaran), A_Est_ (β-O-4 connected with MeGlcA by γ-ester bond), Est (acyl ester), G_Ar_ (guaiacyl), and S_Ar_ (syringyl). For acetylated carbohydrates, for example, the abbreviation X1_2OAc_ refers to xylose C-1 containing acetyl group in the C-2 position.

For more detailed chemical characterization, the non-acetylated starting hemicelluloses were analyzed by 2D HSQC NMR spectroscopy. The HSQC spectra for GGM and GX are presented in Figures 4, 5, respectively. The spectra of samples dissolved in d_6_-DMSO were tentatively identified based on existing data for GGM (Hannuksela and Hervé du Penhoat, 2004; Kim and Ralph, 2014; Berglund et al., 2019), for GX (Teleman et al., 2000, 2002; Rencoret et al., 2012; Kim and Ralph, 2014), for lignin (Liitiä et al., 2003), and for LCC γ-ester (Giummarella et al., 2019), combined with the HSQC-TOCSY NMR spectra (presented in Figures S1A,B). Furthermore, the centrifuged fractions GGM-phe and GX-phe were acetylated prior to analysis to improve solubility in d_6_-DMSO. The HSQC spectra of acetylated GGM-phe and GX-phe side-chain area are presented in Figure 6, for which the signals were identified according to previously published data (Ämmälahti et al., 1998; Qu et al., 2011; Wen et al., 2012; Du et al., 2013). The color codes and symbols used for the chemical structures identified are presented in Figure 7, and the list of peaks is presented in Table S2.

The NMR results support the analysis of carbohydrate composition, and the main carbohydrates shown in Table 2 were also present in the NMR spectra of the starting materials GGM and GX (Figures 4, 5). Thus, the most intensive signals in the spectra of GGM and GX were assigned to Man*p* and Xyl*p*, respectively. Glc*p* could be assigned for both samples, whereas MeGlcA was found only in the spectrum of GX and Gal*p* only in the spectrum of GGM. Interestingly, considering the signal of MeGlcA, for which the MGA4 (see the Figure 7 text for abbreviations used in NMR spectra) does not overlap with other signals, it seems that the threshold limit of this 2D NMR technique prevents the observation of MeGlcA in GGM, in which the relative amount of MeGlcA was lower compared to GX. Both starting hemicelluloses also contained acetates, which are naturally present in wood hemicelluloses, GGM and GX (Sjöström, 1993). The XG1_3OAc_ was identified based on a previous structural characterization of GX of birch, beech, and aspen, because the position of the cross-signal between X1_2,3OAc_ and X1_2OAc_ fits very well with the published data (Teleman et al., 2000, 2002). The abbreviation XG1_3OAc_ refers to the structural element (→ 4)[4O-Me-α-D-Glc*p*A-(1 → 2)][O-Ac-(1 → 3)]-β-D-Xyl*p*-(1 → ). Because the previously published NMR data were obtained in a different solvent (D_2_O), and because there were now more overlapping signals, assignment of the other signals belonging to this xylopyranosyl ring was not possible.

The NMR spectra show also that both starting hemicelluloses contained lignin, for which the signals of β-aryl ether type were the most intensive (Figures 4, 5). Further, the results provide further evidence of the fact that lignin is the phenolic material improving emulsifying/functional properties of PHWE extracts. Because β-O-4 linkage is the most abundant type in native lignin (Sjöström, 1993), the intensity of the signals indicated that the structure of lignin was not extensively degraded but instead preserved during the PHWE process. The other lignin bonding types found in the spectra of the starting hemicelluloses were phenyl coumaran type (β-5), found for both hemicelluloses, and resinol type (β-β), found only for GX. According to the signals in the aromatic region at around 7 ppm, GGM contained only aromatic protons of the guaiacyl type, and GX contained mainly aromatic protons of the syringyl type and a small amount of guaiacyl type protons.

The starting hemicelluloses also contained -CH_2_- protons connected to ester functionality. For GX (Figure 5), the signal at 4.30/62.92 ppm was assigned to the γ-proton of the β-O-4-structure linked to MeGlcA through an ester bond, which is also known as the γ-ester type LCC bond (Li and Helm, 1995; Giummarella et al., 2019). In addition, the signal at 4.30/83.46 ppm was assigned to the β-proton belonging to the same LCC-bonding pattern type, confirming that γ-ester LCC bonds must be rather frequent in GX hemicelluloses produced by the PHWE process. According to model compound studies using smaller synthesized molecules, as well as to those using lignin dehydropolymer (DHP), urunosyl units can migrate to the γ-position (Li and Helm, 1995; Giummarella et al., 2019), and thus it is also possible that LCCs are formed during the PHWE process. Similarly for GGM (Figure 4), the signals at 4.04 and 4.27/63.25–63.4 ppm could be assigned to -CH_2_- protons connected to ester, but because no more of the signals present belonged to the β-O-4-type LCC-bonding pattern, these signals could not be unequivocally identified as originating from lignin. For example, the GGM of *Aloe barbadensis* contains acetyl groups at the C-6 position of Man*p*, which give signals at the same positions in the HSQC spectrum (Campestrini et al., 2013).

As already suggested by the small amount of carbohydrates, the centrifuged fractions GGM-phe and GX-phe (Figure 6) were composed mainly of lignin. The samples were also acetylated prior to analysis in order to improve their solubility in d_6_-DMSO. The typical bonding patterns for lignin were found, and the signals for the β-O-4 bond type were clearly the most intense, similarly to the starting hemicelluloses. For both GGM-phe and GX-phe, the other lignin bonding patterns, the β-5 and β-β structures, were also more clearly identified compared to the NMR spectra of the starting hemicelluloses. The LCC structures could not be clearly identified from these acetylated phenolic fractions, because the signals of γ-esters would in this case overlap with all the acetylated γ-signals in lignin.

### Molar Mass Analysis of Starting Hemicelluloses GGM and GX, Purified Fractions GGM-Pur and GX-Pur, and Precipitated Fractions GGM-Phe and GX-Phe

The results from molar mass analysis by SEC for the starting hemicelluloses GGM and GX and for the purified and phenolic fractions are presented in Table 3. The molar masses of starting GGM, M_w_ of around 7,300 Da, and starting GX, M_w_ of around 3,100 Da, were in a similar range, but slightly lower compared to the previously reported values (Mikkonen et al., 2019). However, the previously obtained results, 8,200 Da for GGM and 4,000 Da for GX, were analyzed in water solutions compared to the DMSO used in this study, which could have affected the results slightly. The molar masses of both purified fractions were similar to those of the starting materials, 7,200 Da for pur-GGM and 3,400 Da for pur-GX.

**Table 3:** Molar masses for starting hemicelluloses, GGM and GX, and the purified and phenolic fractions analyzed by size-exclusion chromatography (SEC) in DMSO.

	**GGM**	**GGM-pur**	**GGM-phe**	**GX**	**GX-pur**	**GX-phe**
M_n_ (Da)	917	949	363	632	710	326
M_w_ (Da)	7,312	7,155	2,769	3,064	3,355	2,472
M_z_ (Da)	20,371	18,276	25,362	6,571	8,243	8,041
M_w_/M_n_	7.97	7.53	7.63	4.85	4.73	7.58

For phenolic fractions containing mainly lignin, the estimated molar masses were lower than those of the starting materials, for GGM-phe significantly lower 2,800 Da and for GX-phe 2,500 Da. Although present knowledge indicates that analysis by SEC gives underestimated molar masses for lignins (Zinovyev et al., 2018), the results show that GGM-phe and GX-phe have similar molar masses but that their molar masses are different from those of the starting hemicelluloses and fractions of purified hemicelluloses. Furthermore, the polydispersity, M_w_/M_n_, for all GGM samples was in the range of 7.5–8.0, although for GX samples the value was higher for GX-phe (7.6) and lower for starting GX (4.9) and GX-pur (4.7). In this respect, the variation of molar masses was similar within the phenolic fractions (GGM-phe and GX-phe) as well as for the GGM starting material and purified fraction GGM-pur, whereas dispersity was slightly lower for starting GX and GX-pur.

### Evaluation of Diffusion Constants by DOSY NMR (Acetylated or Partially Acetylated Samples)

The 2D DOSY results are shown in Table 4. The viscosity corrected value D_c_(GGM) (0.17 × 10^−10^ m^2^s^−1^) is clearly lower than D_c_(GGM-phe) (0.21 × 10^−10^ m^2^s^−1^), D_c_(GX) (0.22 × 10^−10^ m^2^s^−1^), and D_c_(GX-phe) (0.21 × 10^−10^ m^2^s^−1^), the latter three being practically identical. This is in line with the SEC results (Table 3), indicating approximately 7 kDa for GGM-pur and 3 kDa for the others. However, it must be pointed out that the absolute differences in these diffusion coefficients are not large. Because there is a spread in the DOSY-correlations (i.e., all the peaks of the molecule do not appear with the same D_c_ value), it is difficult to pick a representative average value. There are various reasons for the spread, such as possible overlap with other residual entities, success of DOSY fitting, non-optimized diffusion time/diffusion gradient area (in order to achieve sufficient decay), noise, etc. This, combined with the aforementioned small absolute differences, makes these DOSY results indicative at best, but still usable for qualitative purposes. Improvement could be achieved by optimizing the diffusion delays and/or diffusion gradient areas, increasing the number of diffusion steps in DOSY measurement, and increasing number of transients.

**Table 4:** Diffusion coefficients (D_c_) obtained from DOSY NMR of acetylated (or partially acetylated) starting GGM and GX and phenolic fractions GGM-phe and GX-phe.

	**D_c_ (×10^−10^ m^2^s^−1^)**			
**Sample**	**D_**c**_(MM)**	**D_**c**_(DMSO)**	**Correction factor**	**D_**c**_(MM_**Corr**_)**
GGM	0.17	1.30	1.00	0.17
GGM-phe	0.21	1.29	1.01	0.21
GX	0.21	1.39	0.94	0.22
GX-phe	0.22	1.35	0.96	0.21

*D_c_(MM) is the diffusion constant of macromolecule*.

Furthermore, the lignin signals, which do not overlap with other residues, the β-O-4 (1H 5.95 ppm) and ArH (1H 6.65 ppm for GX and 6.97 ppm for GGM), have similar diffusion coefficients compared to carbohydrate signals. This means that lignin has a very similar diffusion coefficient compared to hemicelluloses, which provides further support for covalent association of carbohydrates and lignin.

### Analysis of Phenolic Contents of Starting Hemicelluloses and Phenolic Fractions GGM-Phe and GX-Phe Using Pyrolysis GC/MS

The pyrolysis GC/MS technique (py-GC/MS) was used to evaluate the usefulness of this method for fast characterization of the phenolic content of the starting hemicelluloses and phenolic fractions. Py-GC/MS correlates with the lignin and carbohydrate composition, especially for pulp samples, and can be used fairly reliably for the determination of the S/G ratio, which is the ratio of syringyl and guaiacyl types of units in lignin (del Rio et al., 2002; Ohra-aho et al., 2013, 2018). Thus, a rough estimation of the lignin and carbohydrate content of the starting hemicelluloses GGM and GX and for the lignin-rich residues GGM-phe and GX-phe was made by grouping all the peaks from py-GC/MS and calculating areas of all groups, as presented in Table 5.

**Table 5:** The products found in py-GC/MS of starting hemicelluloses GGM and GX and phenolic fractions GGM-phe and GX-phe.

**Compound**	**GGM**	**GGM-phe**	**GX**	**GX-phe**	**Origin**
Acetic acid			12.34	4.47	Carb
Methyl acetate	11.25				Carb
1-Hydroxypropan-2 one		0.58			Carb
2,3-Pentanedione			0.71		Carb
3-Hydroxypropanal	12.21	1.55	9.76	1.92	Carb
1,4-Butanedial	2.61				Carb
Methyl-2-oxopropanoate	4.79				Carb
3-Furfural			0.50		Carb
Furfural	3.12		6.68	0.43	Carb
2-Furanmethanol	2.73				Carb
2-Butanone			1.42		Carb
Cyclopent-2-ene-1,4-dione	0.44				Carb
2-Methyl-1-cyclopent-2-enone			0.72		Carb
2-(5H)-Furanone	1.60				Carb
2,5-Dihydropyran-6-one	0.82				Carb
2-Hydroxy-2-cyclopenten-1-one	0.88		0.71		Carb
3-Methyl-2-cyclopentenone			0.80		Carb
Phenol	0.83	0.34	0.67		Ar
4-Hydroxy-5,6-dihydro-(2H)-pyran-2-one	1.05		0.59	0.43	Carb
2-Hydroxy-3-methyl-2-cyclopenten-1-one	1.83		1.85		Carb
D-arabinal				0.09	Carb
2,3-Dimethyl-2-cyclopenten-1-one			0.34		Carb
o-Cresol	0.49	0.22	0.29	0.13	Ar
3,4-Dimethyl-2-hydroxy-2-cyclopentanone			0.34		Carb
p-Cresol	0.35	0.47	0.40	0.15	H
Guaiacol	3.32	6.98	1.28	1.50	G
2-Methylpyran-3,4-dione	0.36				Carb
3-Methylguaiacol		0.20	0.14	0.15	G
4-Methylguaiacol	1.34	5.50	0.51	0.69	G
Catechol	1.30				G
5-Hydroxymethylfurfural	0.88				Carb
Methylveratrol	0.14				Ar
3-Methylcatechol	0.57	0.14			Ar
Guaiacyl acetate			0.20		G
3-Methoxycatechol				0.80	Ar
4-Ethylguaiacol	0.79	1.09	0.22	0.24	G
4-Methylcatechol	0.57				Ar
5-Acetoxymethyl-2-furaldehyde	0.39				Carb
4-Vinylguaiacol	3.16	8.34	1.04	1.24	G
Syringol		0.24	6.64	7.37	S
Eugenol	0.67	2.27	0.37	0.41	G
4-Propylguaiacol	0.26	0.33			G
Vanillin	0.64	5.25		1.66	G
*cis*-Isoeugenol	0.32	1.57		0.25	G
4-Methylsyringol			1.23	2.09	S
*trans*-Isoeugenol	1.69	6.54	0.57	0.99	G
Homovanillin	0.52	4.25		0.68	G
Acetovanillone	0.59	3.26		0.90	G
4-Ethylsyringol			0.69	0.66	S
Guaiacylacetone	0.37	1.26		0.20	G
4-Vinylsyringol			4.07	5.31	S
Guaiacylvinylketone				0.53	G
Guaiacylpropenol	0.51	0.88			G
4-Prop-2-enyl syringol^*^			1.55	1.62	S
Dihydroconiferyl alcohol	0.97	3.67			G
Syringaldehyde			0.15	7.56	S
*cis*-Coniferyl alcohol		2.16		0.41	G
4-Propynesyringol			0.31	1.52	S
*cis*-4-Prop-1-enyl syringol^*^			3.80	4.97	S
Homosyringaldehyde				4.38	S
Acetosyringone			0.20	3.59	S
*trans*-Coniferyl alcohol	4.07	20.62		2.63	G
Syringylacetone			0.79	1.10	S
2,6-Dimethoxy-4-[(1e)-1-propenyl]phenyl acetate			0.43		S
Propiosyringone				0.44	S
Syringyl vinyl ketone				1.41	S
Dihydrosinapyl alcohol				0.40	S
*cis*-Sinapyl alcohol			0.47	2.34	S
*trans*-Sinapaldehyde				2.32	S
*trans*-Sinapyl alcohol				13.89	S
Methyldehydroabietate		0.19			RA
9-Oxodehydroabietic acid methyl ester		0.19			RA
**Total Carb**	**44.96**	**2.13**	**36.76**	**7.34**	
Total H	0.35	0.47	0.40	0.15	
Total G	20.52	74.17	4.33	12.48	
Total S	0.00	0.24	20.33	60.97	
**Total Lig (H + G + S)**	**20.87**	**74.88**	**25.06**	**73.60**	
Other units	2.60	1.08			
**S/G ratio**			**4.70**	**4.89**	

*The compounds originated from carbohydrates (Carb), p-hydroxyphenyl (H), guaiacyl (G), or syringyl-type (S) lignin units, other aromatic units (Ar), or rosin acids (RA)*.

* *May contain areas of two signals identified to the same compound by GC-MS. The significance to the total value is 1% or less*.

*The values are presented as percentages of the peak area compared to the total peak area*.

The results were then compared to the acid methanolysis followed by GC analysis of total carbohydrates (in Table 2). Assuming that the starting hemicelluloses and phenolic fractions contained only hemicelluloses and lignin, the results of the methods should be compatible. However, the carbohydrate contents from determined py-GC/MS were much lower compared to the results obtained from acid methanolysis-GC method. On the other hand, the lignin content from py-GC/MS seemed fairly reasonable when compared to total carbohydrate content from acid methanolysis. When the total carbohydrate content from acid methanolysis and lignin content from py-GC/MS were summed, the total content (lignin + carbohydrates) was 94.34% for starting GGM, 100.01% for GGM-phe, 86.54% for starting GX, and 91.73% for GX-phe. According to previous results, the py-GC/MS-analysis of carbohydrate content is not necessarily reliable for comparing samples containing different carbohydrates (Ohra-aho et al., 2018), which probably affected the results presented here as well. However, for the rough evaluation of lignin content in samples of hemicelluloses, the method could be suitable, and it could provide estimations of the carbohydrate content in an indirect way.

The S/G ratio of the starting GX and GX-phe samples was very similar (4.70 and 4.89, respectively; Table 5). The reliability of analyzing the S/G ratio by py-GC/MS has been shown for eucalyptus samples (Ohra-aho et al., 2013), and the method is most likely valid for GX hemicelluloses. A recently published S/G ratio for birch wood from Sweden was 3.25 (Wang et al., 2018). Although the results are not necessarily comparable for samples from different wood materials, the S/G ratio obtained for lignin associated with GX seems fairly high, also taking into consideration the results obtained for other hardwood species. For example, in another study of eucalyptus samples, the S/G ratio was 1.9–3.1 (Ohra-aho et al., 2013).

### Analysis and Quantitation of Small Extractable Phenolic Compounds From Phenolic Fractions GGM-Phe and GX-Phe—Vanillin and Syringaldehyde as Indicators in Lignin Participating in Formation of Emulsions

A previous study showed that certain types of extractable small phenolic compounds of PHWE GGM concentrate were adsorbed on the oil droplets of rapeseed oil emulsions (Lehtonen et al., 2018). It was then assumed that LCC structures composed of phenolic and carbohydrate residues would improve the emulsification and stabilization ability of PHWE hemicelluloses. We now assume also that the extractable phenolic compounds would be associated with lignin present in the samples. The phenolic fractions GGM-phe and GX-phe were extracted and analyzed with UPLC, and the main peaks were identified with LC-MS and then quantified using corresponding standards.

The main small phenolic compounds identified according to LC-MS were vanillin (in both GGM and GX) and syringaldehyde (only in GX). The amounts found in GGM-phe and GX-phe are shown in Table 6. Both compounds were found mainly in the ethanol soluble fractions; GX was not even precipitated during extractions. The amounts of compounds dissolved in neutral solvent and additionally acid hydrolyzed were very similar. Clearly, the highest amount of these compounds was released by base hydrolysis.

**Table 6:** The amounts obtained from UPLC analysis of main small extractable phenolic compounds, vanillin and syringaldehyde, which were identified as the main products extracted from the phenolic residues GGM-phe and GX-phe.

**Sample**	**Extraction method**	**Vanillin (μg/g)**	**Syringaldehyde (μg/g)**	**S/G ratio**
GGM-phe	Ethanol soluble	818 ± 109	ND	
	Ethanol soluble + acid hydrolysis	995 ± 156	ND	
	Ethanol soluble + base hydrolysis	6,148 ± 635	ND	
	Ethanol precipitated + acid hydrolysis	28 ± 5	ND	
	Ethanol precipitated + base hydrolysis	101 ± 26	ND	
GX-phe	Ethanol soluble	255 ± 21	760 ± 55	2.98
	Ethanol soluble + acid hydrolysis	313 ± 40	948 ± 122	3.02
	Ethanol soluble + base hydrolysis	1,603 ± 23	7,677 ± 170	4.79

*ND, not detected*.

The total amount of vanillin and syringaldehyde extracted was <0.1 m-%, meaning that the amount was still much lower considering the starting hemicelluloses. However, the classification of vanillin and syringaldehyde would fit that of hydroxycinnamic acids (OHCs) in terms of the previously used classification (Lehtonen et al., 2018). On the other hand, ethanol soluble phenols belonging to OHCs were found solely adsorbed in the oil of the emulsion, which means that vanillin bound to GGM-phe and syringaldehyde bound to GX-phe also participate in the formation of emulsions. Furthermore, because these compounds are clearly mainly covalently bound to the phenolic fractions containing lignin, it is also likely that lignin is involved in the formation and stabilization of emulsions.

The S/G ratio of syringaldehyde and vanillin extracted and base-hydrolyzed from GX-phe was 4.79, which is very close to the value obtained from py-GC/MS for the whole lignin. Although the values could be similar by coincidence, it is more likely that the similar S/G ratio obtained reflects the presence of lignin adsorbed with hemicelluloses to the surface of emulsion droplets. Because we have not thus far been able to completely release hemicelluloses adsorbed on rapeseed oil droplets, this quantitation by UPLC is by far the best method for identifying the presence of lignin in emulsions stabilized with PHWE hemicelluloses, and it can be used to tag on lignin associated with hemicelluloses.

### Properties of Hemicelluloses Affecting the Physical Properties and Stability of Emulsions

The results regarding the droplet-size distribution of emulsions (Figure 1, Table 1) can be explained by the presence of lignin. For fresh emulsions prepared using purified GGM-pur and GX-pur fractions, the D[3,2] values were smaller compared to starting GGM and GX. It is reasonable to assume that lignin's participation in the formation of oil droplets would increase their size.

Regarding the physical stability of emulsions, the droplet size increased faster during the storage of emulsions stabilized with GGM-pur and GX-pur compared to emulsions stabilized with the starting hemicelluloses. This indicates that the presence of lignin stabilizes the physical structure of emulsions. For PHWE GGM, it was recently demonstrated that the mixed mechanism involves Pickering stabilization with interfacial adsorption of GGM, which are probably associated with lignin (Valoppi et al., 2019a). In addition, the bimodal distribution of GX into smaller and larger droplets was less enhanced in the presence of lignin.

It is evident that lignin, as a natural antioxidant, is also responsible for the improved oxidative stability of emulsions. However, oxidation of phenolic compounds may also change their chemical structure, which could further induce structural changes and affect the physical stability of emulsions. The presence of LCC bonds was evident from the NMR spectrum of starting GX, and the γ-ester structures found could be at least partially responsible for the functional properties of PHWE hemicelluloses, allowing the lignin part anchor to the oil droplet surface, as hypothesized previously (Lehtonen et al., 2018). In this case, it is not necessary to debate whether the LCCs are derived from the starting wood material or produced during the PHWE process; the essential point is the excellent functional properties of hemicelluloses produced by the PHWE process.

## Conclusions

We showed that phenolic structures, which were partially removed from both GGM- and GX-rich wood extracts by using centrifugal forces, played a key role in emulsion stability. The proportions, chemical compositions, and molar masses of the phenolic-rich fraction varied between GGM and GX hemicelluloses. Complementary chemical characterization of centrifuged materials showed that the phenolic-rich fraction contained mainly native lignin and a small amount of carbohydrates.

Using various approaches, the results confirmed that this phenolic-rich fraction improved both the physical and the oxidative stability of emulsions stabilized with PHWE extracts. The antioxidative properties of phenolic compounds coextracted with hemicelluloses may also be interlinked with the physical stability of emulsions. Furthermore, NMR analysis confirmed the presence of a high concentration of γ-ester type LCCs, which could explain the excellent emulsifying capacity of PHWE hemicelluloses. Both GGM and GX produced emulsions with high physical and oxidative stability, although the emulsions had slightly different types of characteristics depending on the source of hemicellulose. The results also showed that in order to achieve desired emulsifying properties, the total removal of lignin is not advisable; in fact, it introduces unnecessary complexities into the PHWE biorefining process.

## Data Availability Statement

All datasets generated for this study are included in the article/Supplementary Material.

## Author Contributions

KM planned and received funding for the project. ML mainly designed the experimental plan, with expertise in wood chemistry, with the help of KM (emulsions and hemicelluloses) and FV (emulsions, fractionation of hemicelluloses by centrifugal forces). ML performed and analyzed the 2D HSQC NMR of hemicelluloses, did part of the practical work during the preparation and characterization of emulsions, performed and analyzed the phenolic extraction procedures by UPLC and LC-MS, and assumed the main responsibility for writing the manuscript and interpreting the data. PK provided the materials for the study as well as knowledge about PHWE hemicelluloses and the process. VJ analyzed the carbohydrate content under the guidance of ML. VJ also contributed to the preparation and characterization of emulsions. SH designed and performed the DOSY NMR analysis, provided technical support during NMR analysis, and contributed to the writing of the experimental details of NMR for the manuscript. NM contributed to the calibration of SEC data and determination of molar masses. All authors read and commented on the manuscript.

### Conflict of Interest

The authors declare that the research was conducted in the absence of any commercial or financial relationships that could be construed as a potential conflict of interest.
