# Supplementary material for: Lignin-Rich PHWE Hemicellulose Extracts Responsible for Extended Emulsion Stabilization
Source: Front Chem. 2019 Dec 17;7:871. doi: 10.3389/fchem.2019.00871 (PMC6927942; doi:10.3389/fchem.2019.00871)
Supplement: Supplementary file 1 [file Table_1.DOCX]

Supplementary Material

# Droplet-size distribution measurements, all results

**Table S1.** Measured data obtained from droplet-size distribution measurements. The D[3,2] and D[4,3] are the surface and volume weighted mean diameters. The D10, D50 and D90 values mean that 10%, 50% and 90% of droplets are smaller compared to that value.

| **Sample** | **D[3,2]/**  **(μm)** | **D[4,3]/**  **(μm)** | **D(10)/**  **(μm)** | **D(50)/**  **(μm)** | **D(90)/**  **(μm)** |
| --- | --- | --- | --- | --- | --- |
| GGM week 0 | 0.152 | 0.552 | 0.0700 | 0.237 | 1.05 |
| GGM week 1 | 0.177 | 1.30 | 0.0818 | 0.255 | 1.44 |
| GGM week 2 | 0.176 | 1.97 | 0.0810 | 0.255 | 1.82 |
| GGM week 4 | 0.193 | 2.16 | 0.0894 | 0.271 | 2.93 |
| GGM week 6 | 0.202 | 2.88 | 0.0929 | 0.28 | 4.12 |
| GGM week 8 | 0.201 | 4.27 | 0.0916 | 0.281 | 5.42 |
| GGM week 11 | 0.207 | 8.09 | 0.0928 | 0.289 | 10.1 |
| GGM week 13 | 0.212 | 6.95 | 0.0946 | 0.294 | 11.5 |
| GX day 0 | 0.137 | 1.07 | 0.0572 | 0.259 | 4.02 |
| GX week 1 | 0.169 | 2.56 | 0.0718 | 0.299 | 7.11 |
| GX week 2 | 0.171 | 3.1 | 0.0725 | 0.301 | 9.44 |
| GX week 4 | 0.21 | 4.14 | 0.0873 | 0.343 | 15.1 |
| GX week 6 | 0.243 | 4.23 | 0.0997 | 0.376 | 15.3 |
| GX week 8 | 0.249 | 4.7 | 0.1 | 0.393 | 16.4 |
| GX week 11 | 0.265 | 6.47 | 0.105 | 0.427 | 19.8 |
| GX week 13 | 0.26 | 5.35 | 0.104 | 0.413 | 17.9 |
| GGM-pur day 0 | 0.117 | 0.215 | 0.0555 | 0.187 | 0.415 |
| GGM-pur week 1 | 0.126 | 0.857 | 0.0585 | 0.199 | 0.561 |
| GGM-pur week 2 | 0.144 | 1.13 | 0.0677 | 0.214 | 4.18 |
| GGM-pur week 4 | 0.18 | 1.56 | 0.0835 | 0.243 | 6.61 |
| GGM-pur week 6 | 0.202 | 2.94 | 0.0924 | 0.266 | 7.76 |
| GGM-pur week 8 | 0.211 | 5.1 | 0.0949 | 0.278 | 8.47 |
| GGM-pur week 11 | 0.219 | 3.4 | 0.0980 | 0.286 | 8.17 |
| GGM-pur week 13 | 0.226 | 4.87 | 0.100 | 0.295 | 8.85 |
| GX-pur day 0 | 0.116 | 1.40 | 0.0479 | 0.205 | 6.16 |
| GX-pur week 1 | 0.242 | 4.39 | 0.0969 | 0.353 | 12.3 |
| GX-pur week 2 | 0.263 | 5.67 | 0.103 | 0.402 | 16.8 |
| GX-pur week 4 | 0.313 | 6.80 | 0.116 | 0.599 | 19.5 |
| GX-pur week 6 | 0.32 | 5.67 | 0.120 | 0.553 | 15.4 |
| GX-pur week 8 | 0.344 | 9.01 | 0.124 | 4.13 | 21.2 |
| GX-pur week 11 | 0.356 | 8.75 | 0.127 | 4.19 | 23.1 |
| GX-pur week 13 | 0.343 | 9.58 | 0.122 | 3.94 | 24.6 |

# List of NMR assignments

**Table S2.** List of NMR assignments, which were evaluated using various sources and complemented by using HSQC-TOCSY data (references used are found in the article). Ac = acetylated.

|  | δH/δC (ppm) | | | |
| --- | --- | --- | --- | --- |
| Assignment | **GGM** | **GX** | **GGM-phe (Ac)** | **GX-phe (Ac)** |
| A: β-O-4 α | 4.73/70.67 | 4.87/71.93 | 5.93;5.97/73.30;73.96 | 5.92/73.82 |
| A: β-O-4 β | 4.23/83.57 | 4.11/85.86 | 4.84/78.41 | 4.63/79.69 |
| A: β-O-4 γ |  |  |  |  |
| A: β-O-4 γ’ |  |  | 4.11/62.34 | 4.27/62.08 |
| B: β-β α |  | 4.62/85.13 | 4.77/84.19 | 4.75/84.66 |
| B: β-β β |  | 3.05/53.52 | 3.09/53.68 | 3.11/53.51 |
| B: β-β γ |  | 3.77/70.18 | Man4 overlaps | 3.88/71.08 |
| B: β-β γ’ |  | 4.16/70.90 | 4.17/70.82 | 4.22/71.03 |
| C: β-5 α | 5.43/86.55 | 5.41/87.01 | 5.58/86.65 | 5.52/86.97 |
| C: β-5 β | 3.46/52.72 |  | 3.77/49.03 | 3.80/49.22 |
| C: β-5 γ |  |  |  |  |
| C: β-5 γ’ |  |  | 4.34/64.46 |  |
| A_Est_: β |  | 4.30/83.46 |  |  |
| A_Est_: γ |  |  |  |  |
| A_Est_: γ’ |  | 4.30/62.92 |  |  |
| Est: γ | 4.04/63.40 |  |  |  |
| Est: γ’ | 4.27/63.25 |  |  |  |
| -OCH_3_ | 3.73/55.08 | 3.74/55.73 | 3.76/55.45 | 3.75/55.54 |
| -OAc | 2.03/50.54 | 1.99/20.65 |  |  |
| Man1 | 4.53/100.20 |  | 4.93/96.32 |  |
| Man1_2OAc_ | 4.67/99.25 |  |  |  |
| Man1_3OAc_ | 4.75/98.63 |  |  |  |
| Man2 | 3.76/69.26 |  | 5.27/68.55 |  |
| Man2_2OAc_ | 5.28/70.33 |  |  |  |
| Man2_3OAc_ | 3.84/67.74 |  |  |  |
| Man3 | 3.45/71.37 |  | 5.17/68.83 |  |
| Man3_3OAc_ | 4.73/70.86 |  |  |  |
| Man4 | 3.60/76.95 |  | 3.85/71.54 |  |
| Man5 | (3.28-3.37/74.51-74.91) |  | 3.69/71.24 |  |
| Man6 | 3.53/60.05 |  |  |  |
| Man6’ | 3.62/59.99 |  |  |  |
| Glc1 | 4.27/101.68 | 4.17/103.15 | 4.72/99.52 |  |
| Glc2 | 3.04/72.48 | 2.87/72.83 | 4.54/70.62 |  |
| Glc3 |  |  | 5.08/71.68 |  |
| Glc4 | 3.39/79.04 | 3.46/76.52 | 3.78/74.32 |  |
| Glc5 | 3.11/76.27 | 3.07/76.10 | Man4 overlaps |  |
| Glc6 | 3.53/60.05 |  |  |  |
| Glc6’ | 3.62/59.99 |  |  |  |
| MeGlcA1 |  | 5.08/96.88 |  |  |
| MeGlcA2 |  | 3.19/70.23 |  |  |
| MeGlcA3 |  | 3.44/71.53 |  |  |
| MeGlcA4 |  | 3.05/82.07 |  |  |
| Xyl1 |  | 4.27/101.65 | Glc1 overlaps | 4.71/99.54 |
| Xyl1_2OAc_ |  | 4.51/99.38 |  |  |
| Xyl1_3OAc_ |  | 4.39/101.38 |  |  |
| Xyl1_2,3OAc_ |  | 4.71/98.87 |  |  |
| Xyl1_α_R |  | 4.85/92.07 |  |  |
| Xyl1_β_R |  | 4.22/97.33 |  |  |
| Xyl-(Glc)-1_3OAc_ |  | 4.59/101.2 |  |  |
| Xyl2 |  | 3.04/72.40 | Glc2 overlaps | 4.52/70.41 |
| Xyl2_2OAc_ |  | 4.50/73.20 |  |  |
| Xyl2_2,3OAc_ |  | 4.61/70.93 |  |  |
| Xyl3 |  | 3.22/73.87 | 4.95/71.68 | 4.95/71.68 |
| Xyl3_3OAc_ |  | 4.80/74.70 |  |  |
| Xyl3_2,3OAc_ |  | 4.98/72.47 |  |  |
| Xyl4 | 3.50/75.16 | 3.50-3.61/  75.32-75.34 | Glc4 overlaps | 3.78/74.70 |
| Xyl4_3OAc_ |  | 3.76/75.06 |  |  |
| Xyl5 | 3.17/62.90 | 3.16/63.01 | 3.32/62.05 | 3.31/62.11 |
| Xyl5’ | 3.87/62.90 | 3.90/62.95 | -CH_2_O-area | 3.89/62.13 |
| Xyl5NR | 3.06/65.60 | 3.05/65.66 |  |  |
| Xyl5NR’ | 3.71/65.69 | 3.70/65.60 |  |  |
| Gal1 | 4.26/105.09 |  |  |  |
| Gal1_β_R | 4.22/97.22 |  |  |  |
| G_Ar_-2 | 6.98/110.91 | 6.92/110.40 |  |  |
| G_Ar_-5 | 6.69-6.91/  114.56-114.96 | 6.68-6.91/  114.73 |  |  |
| G_Ar_-6 | 6.76/118.48 | 6.74/118.69 |  |  |
| S_Ar_-2,6 |  | 6.69/103.94 |  |  |

# HSQC-TOCSY NMR spectra of nonacetylated GGM and GX

**Figure S1a.** Magnification of HSQC-TOCSY NMR spectrum of GGM (nonacetylated) in d_6_-DMSO.

**Figure S1b.** Magnification of HSQC-TOCSY NMR spectrum of GX (nonacetylated) in d_6_-DMSO.

# DOSY NMR spectra

**Figure S2a.** DOSY NMR of GGM (partially acetylated).

**Figure S2b.** DOSY NMR of GGM-phe (acetylated).

**Figure S2c.** DOSY NMR of GX (partially acetylated).

**Figure S2d.** DOSY NMR of GX-phe (acetylated).

# Pyrograms from py-GC/MS

**Figure S3a.** Pyrogram of GGM from py-GC/MS analysis.

**Figure S3b.** Pyrogram of GGM-phe from py-GC/MS analysis.

**Figure S3c.** Pyrogram of GX from py-GC/MS analysis.

**Figure S3d.** Pyrogram of GX-phe from py-GC/MS analysis.

# LC-MS analysis of extracted small phenolic compounds from GGM-phe and GX-phe

**Figure S4a.** The signal identified as vanillin in LC-MS analysis of GGM-phe.

**Figure S4b.** The ESI-MS spectrum from the signal identified as vanillin in LC-MS analysis of GGM-phe.

**Figure S4c.** The results from LC-MS analysis of GX-phe. The main signals at 280 nm (the upper chromatogram) are from vanillin (rt 11.72 min) and syringaldehyde (rt 13.74 min).

**Figure S4d.** The ESI-MS spectrum from the signal identified as syringaldehyde in LC-MS analysis of GX-phe.
